# Residual β activity of particulate ^234^Th as a novel proxy for tracking sediment resuspension in the ocean

**DOI:** 10.1038/srep27069

**Published:** 2016-06-02

**Authors:** Wuhui Lin, Liqi Chen, Shi Zeng, Tao Li, Yinghui Wang, Kefu Yu

**Affiliations:** 1 School of Marine Science, Guangxi University, Nanning, China; 2 Key Laboratory of Global Change and Marine-Atmospheric Chemistry, Third Institute of Oceanography, State Oceanic Administration, Xiamen, China; 3 Department of Engineering Physics, Tsinghua University, Beijing, China; 4 Ocean University of China, Qingdao, China

## Abstract

Sediment resuspension occurs in the global ocean, which greatly affects material exchange between the sediment and the overlying seawater. The behaviours of carbon, nutrients, heavy metals, and other pollutants at the sediment-seawater boundary will further link to climate change, eutrophication, and marine pollution. Residual β activity of particulate ^234^Th (RA_P234_) is used as a novel proxy to track sediment resuspension in different marine environments, including the western Arctic Ocean, the South China Sea, and the Southern Ocean. Sediment resuspension identified by high activity of RA_P234_ is supported by different lines of evidence including seawater turbidity, residence time of total ^234^Th, Goldschmidt’s classification, and ratio of RA_P234_ to particulate organic carbon. A conceptual model is proposed to elucidate the mechanism for RA_P234_ with dominant contributions from ^234^Th-^238^U and ^212^Bi-^228^Th. The ‘slope assumption’ for RA_P234_ indicated increasing intensity of sediment resuspension from spring to autumn under the influence of the East Asian monsoon system. RA_P234_ can shed new light on ^234^Th-based particle dynamics and should benefit the interpretation of historical ^234^Th-^238^U database. RA_P234_ resembles lithophile elements and has broad implications for investigating particle dynamics in the estuary-shelf-slope-ocean continuum and linkage of the atmosphere-ocean-sediment system.

Active biogeochemical processes associated with sediment resuspension have occurred in the global ocean^1^. The interactions between sediment and seawater play an important role in the burial of materials and their resupplementation into the overlying water column, which greatly affect the carbon cycle, nutrients, trace metals, and other pollutants. The bottom nepheloid layer occurs at the boundary between sediment and seawater and was widely studied in the GEOSECS-JGOFS-GEOTRACES era^2^. Although the bottom nepheloid layer can be identified by physical^3^, biological^4^, chemical^5^, and geological parameters^6^, the quantitative particle dynamic processes have been investigated by ^234^Th/^238^U disequilibrium method with particulate ^234^Th, dissolved ^234^Th, activity ratio of ^234^Th to ^238^U, activity ratio of particulate ^234^Th to dissolved ^234^Th, and residence time of ^234^Th^6^,^7^,^8^,^9^,^10^.

The ^234^Th/^238^U disequilibrium method is on the basis of the distinct behaviours of ^234^Th and ^238^U in seawater (the particle-reactive ^234^Th and conservative ^238^U). This method is used to quantify the particle process of sediment resuspension^7^,^9^. However, photosynthesis generally occurs alongside sediment resuspension on the shallow continental shelf due to the penetration of sun-light into the full seawater column. The ^234^Th/^238^U disequilibrium method reflects the integrated results of all particle processes in seawater and cannot distinguish sediment resuspension from photosynthesis. Additionally, sediment resuspension enhances the scavenging of ^234^Th and probably leads to the overestimation of export flux of ^234^Th on the shallow continental shelf. Owing to the shortcomings of ^234^Th/^238^U disequilibrium method, Residual β activity of particulate ^234^Th (RA_P234_) is proposed for the first time to track sediment resuspension.

RA_P234_ derived from the second counting rate of particulate ^234^Th could be a powerful complement to ^234^Th/^238^U disequilibrium method in tracking sediment resuspension from low- to high-latitude oceans, including the western Arctic Ocean, the South China Sea, and the Southern Ocean. Seawater turbidity, residence time of total ^234^Th, and the ratio of RA_P234_ to particulate organic carbon (POC) were also measured to support the occurrence of sediment resuspension. The mechanism and conceptual model of RA_P234_ are represented and illustrated. This new definition of RA_P234_ is analogous to that of gross β in drinking water^11^. RA_P234_ is a sensitive proxy for distinguishing sediment resuspension from photosynthesis and for indicating the intensity of sediment resuspension without any additional sampling and measurement. Although further work needs to be conducted, RA_P234_ behaves in a similar manner to lithophile elements and can be a novel approach to investigate quantitative particle dynamics in the estuary-shelf-slope-open ocean continuum.

## Results and Discussion

### Abnormally high activity of RA_P234_

**Figure 1:**
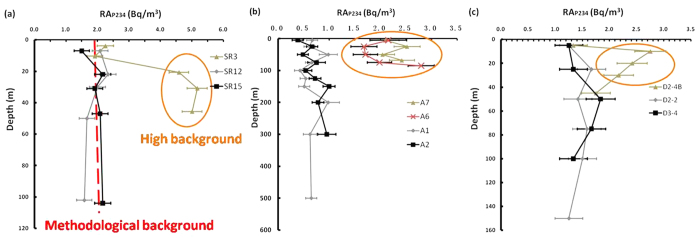
Vertical profiles of RA_P234_ in the western Arctic Ocean (**a**), South China Sea (**b**), and Southern Ocean (**c**). High Activity of RA_P234_ was highlighted with brown circle.

The second counting rate of particulate ^234^Th is generally overlooked due to its constant value of 0.3–0.4 counts per minute (cpm)^12^,^13^,^14^. In this study, RA_P234_ was calculated (see Appendix A1–A3), and data at selected stations are depicted in Fig. 1. High activity of RA_P234_ was commonly observed from low- to high-latitude oceans.

In the western Arctic Ocean (Fig. 1a), RA_P234_ did not vary with depth at deep stations (SR12 and SR15) with a mean activity of 1.95 ± 0.28 Bq/m^3^. However, RA_P234_ increased with depth on the continental shelf (e.g., SR3). The layers (SR3) can be divided into upper layers (2.08 ± 0.24 Bq/m^3^) and deep layers (4.92 ± 0.30 Bq/m^3^), which were separated by the halocline at a depth of 10m according to the salinity profile. RA_P234_ of the upper layers at SR3 was comparable with that of stations SR12 and SR15, while RA_P234_ of the deep layers at SR3 was significantly higher than that of SR12 and SR15.

This high RA_P234_ value could be qualitatively related to sediment resuspension and is used to determine the impact of sediment resuspension on export flux of ^234^Th without any additional sampling and measurement. The layer with a high RA_P234_ value must be screened out during the integration of ^234^Th and POC export fluxes, because sediment resuspension can bias ^234^Th and POC fluxes related to photosynthesis. The finer grain size of the surface sediment as well as intensive hydrodynamics were observed at station SR3 in shallow waters, which favoured sediment resuspension^15^,^16^. Additionally, the high particulate ^210^Pb and deficit of ^234^Th to ^238^U had also been attributed to sediment resuspension on the continental shelf of the Chukchi Sea^10^,^17^.

In the South China Sea (Fig. 1b), high activity of RA_P234_ was evident on the continental shelf (A6 and A7) outside the Pearl River Estuary during autumn. The average RA_P234_ activities for coastal stations (A6 and A7) and open ocean stations (A1 and A2) were 2.16 ± 0.37 Bq/m^3^ and 0.68 ± 0.19 Bq/m^3^, respectively. This region was significantly affected by monsoon winds, especially during autumn. Sediment resuspension was stimulated under the influence of strong winds and shallow depth on the continental shelf, which had previously been demonstrated via the activity ratio of particulate ^234^Th to dissolved ^234^Th^8^.

As for the Southern Ocean (Fig. 1c), sediment resuspension also occurred at coastal station (D2-4B) due to active hydrodynamics and shallow depths near Elephant Island. The ‘Island Effect’ had been demonstrated to be a significant process for providing iron from the sediment to stimulate primary production in this ‘High Nutrient Low Chlorophyll’ region^18^. The average RA_P234_ value at station D2-4B (2.27 ± 0.42 Bq/m^3^) was higher than that of D2-2 and D3-4 (1.46 ± 0.20 Bq/m^3^) in the open ocean, which could be used as a novel approach to identify sediment resuspension.

### Mechanism of RA_P234_

The potential radionuclides associated with high RA_P234_ activity and suspended particles in the seawater can be classified into external and internal radionuclides. The external radionuclides associated with suspended particles refer to the surface-bound radionuclides with high particle reactivity. The radionuclides and their activities in natural seawater have previously been compiled^19^ and can be classified into low and high particle reactivity (Table 1) according to their particle-seawater distribution coefficient (K_d_)^20^. A high K_d_ indicates high particle reactivity. Other artificial radionuclides with short half-lives are not considered due to a lack of nuclear facilities in our sampling region. Otherwise, radionuclides, such as ^91^Y, ^152^Eu, etc., should be taken into account when nuclear fuel reprocessing facilities are in operation near this sea area^21^.

Although the components of suspended particles, including POC, lithogenic materials, biogenic inorganic materials, and hydrogenous materials, display distinct affinity for radionuclides^22^, the requirements for major external radionuclides are high activity in seawater and high K_d_. The high activity of ^234^Th with high particle reactivity results in the direct measurement of surface-bound particulate ^234^Th without additional radiochemical separation. The β counts contributed by ^210^Pb/^210^Bi and other radionuclides with low activity and low energy β particles were shielded by a layer of Mylar film and two layers of aluminium foil (16 mg/cm^2^)^23^,^24^, to prevent external contributions to the β counting^21^. Therefore, the dominant external radionuclide associated with the suspended particles is ^234^Th after sample collection.

Surface-bound ^234^Th with a short half-life (24.1 days) on the suspended particles was unsupported by its parent radionuclides ^238^U, which remains in the seawater due to low K_d_. RA_P234_ was measured over 120 days after sampling. This surface-bound ^234^Th on external particles will decay away. Consequently, the unsupported ^234^Th adsorbed on external particles should not contribute to RA_P234_.

In our study, only RA_P234_ derived from the second counting rate of particulate ^234^Th was investigated. Low activity and low K_d_ of radium in seawater lead to extremely low activity of radium for adsorption onto external suspended particles. Radium and its progenies, such as ^224^Ra^25^, should not contribute to RA_P234_ via surface adsorption. Additionally, to our knowledge, there is no tectonically active region on the continental shelf of the Chukchi Sea to provide high radium activity^26^. Therefore, the radionuclides with low K_d_ in seawater, such as radium, should not significantly contribute to RA_P234_.

The internal radionuclides of suspended particles were more complicate than the external radionuclides. The suspended particles can be classified into terrigenous and biogenic particles in order to analyse the internal radionuclides.

In the bottom nepheloid layer, terrigenous particles resuspended from marine sediment could reach 70%^27^. The concentration of suspended particles in the bottom layer of seawater reached values of up to 9.5 mg/L on the continental shelf of the western Arctic Ocean during the 5th Chinese National Arctic Research Expedition (CHINARE-5), which was significantly higher than that of the upper seawater and indicated the occurrence of sediment resuspension. High concentrations of suspended particle material were also observed for bottom seawater in the western Arctic Ocean^10^. The activity, emitting particle type with energy, and the yield for radionuclides in the marine sediment are presented in Table 2. The activities of some radionuclides are consistent with the limiting direct measurement in the Chukchi Sea^28^. Some α-particle-emitting radionuclides are also presented, because the daughter radionuclides supported by these α-particle-emitting radionuclides could contribute to the β count, such as ^226^Ra and its daughter radionuclides. Therefore, an exhaustive overview of the radionuclides in biogenic and terrigenous particles will benefit comprehensive understanding.

The requirements of the major contributors to internal radionuclides include high activity, high energy of β particles, and high yield. ^234m^Pa, the daughter radionuclide of ^234^Th, emits β particles with a maximum energy of 2.28 MeV (Table 2). The small-volume technique via β counting of ^234^Th is on the basis of ^234m^Pa measurement. The lower energy β particles from other radionuclides were significantly shielded during source preparation with a layer of Mylar film and two layers of aluminium foil^23^.

^234^Th, the parent radionuclide of ^234m^Pa, is supported by the primordial radionuclide ^238^U in the minerals derived from the crust. ^238^U in marine sediments was reported to have a mean activity of 50 Bq/kg in the Chukchi Sea^28^. Terrigenous particles from marine sediment via sediment resuspension can reach 70% in the bottom nepheloid layer^27^. It has also been reported that particulate ^238^U can even reach 95% of total ^238^U due to sediment resuspension^29^. Therefore, ^234^Th supported by ^238^U is probably the dominant contribution to internal radionuclides, especially on the shallow continental shelf with active hydrodynamics.

Although the β energy of ^90^Y was the same as ^234m^Pa (Table 2), the activity of particulate ^90^Y was very low in seawater. The anthropogenic radionuclide of ^90^Sr, the parent radionuclide of ^90^Y, was mainly from global fallout in the Chukchi Sea. Although direct measurement for ^90^Sr had not, to our knowledge, been reported, a fingerprint activity ratio of ^90^Sr to ^137^Cs had a value of 0.63 for global fallout^30^. Combining the reported activity of ^137^Cs with an average value of 2.0 Bq/kg for surface sediment in the Chukchi Sea^28^, the activity of ^90^Sr was about 1.3 Bq/kg, which was less than 5% of that of ^234m^Pa supported by ^234^Th/^238^U. Therefore, the β counting rate contributed by ^90^Y should be minor.

^212^Bi, the progeny of ^228^Th-^232^Th, has the β energy of 2.25 MeV, with a yield of 48.4%. The β energy of ^212^Bi is also close to that of ^234m^Pa with similar detector efficiency. The activity of ^232^Th was generally comparable with that of ^238^U in the marine sediment derived from the crust^31^,^32^. High activity of ^228^Th in bottom layer seawater had previously been directly measured as a result of sediment resuspension in the Baltic Sea^31^. Therefore, ^212^Bi supported by ^228^Th-^232^Th should be considered when sediment resuspension occurs due to its high activity, β energy, and yield.

Although the existence of ^40^K, ^226^Ra, and ^210^Pb had been confirmed by γ spectrometry for bottom seawater in the northeast Atlantic Ocean^33^, these radionuclides and their progenies have lower β particle energies. Their contribution to β counting rate should be minimal due to the shielding effect of aluminium foil^24^.

Biogenic particles make a major contribution to suspended particles in the upper ocean when photosynthesis occurs. The biotas make preferential use of low atomic number elements, such as C, N, P, S and others. Many radionuclides with high atomic numbers are not essential elements for these biotas. Typical radionuclides found in marine biotas are shown in Table 3. The dominant radionuclide amongst the marine biotas is ^40^K, the activity of which is two orders of magnitude greater than that of the other radionuclides. The shield effect of aluminium foil during source preparation of particulate ^234^Th limits the contribution by ^40^K to RA_P234_ due to low β energy. Therefore, the activity of RA_P234_ was low in the euphotic layer due to a major fraction of suspended particles from photosynthesis.

The dominant radionuclides contributing to the high activity of RA_P234_ in bottom seawater are likely to be ^234^Th-^238^U and ^212^Bi-^228^Th in the particles resuspended from marine sediments. RA_P234_ was re-measured three times 120 days after the sampling date to check its stability. It indicated that RA_P234_ is constant due to the long half-lives of ^238^U and ^228^Th-^232^Th and the relatively enclosed environment of the crystal lattice in the mineral derived from marine sediments that constrain any deficit or ingrowth process of the daughter-parent radionuclides.

However, the exact percentage of ^234^Th-^238^U and ^212^Bi-^228^Th for RA_P234_ was not obtained in our study due to limitations on the volume of seawater available. Only 4–8 L of seawater was sampled on the continental shelf for ^234^Th analysis. The phenomenon, abnormally high activity of RA_P234_, was found during data analysis. Although the radionuclides of ^234^Th-^238^U and ^212^Bi-^228^Th could be checked by α-spectrometry and γ-spectrometry, a large amount of seawater (>100 L) should be sampled due to the lower detector efficiency of γ-spectrometry (<1%) and α-spectrometry (10%~20%) relative to that of β-counter in this study (47 ± 2%). Chemical recovery should also be considered via α-spectrometry. RA_P234_, which combines the dominant β signal from ^234^Th-^238^U and ^212^Bi-^228^Th, is sensitive enough to indicate sediment resuspension with small volume of seawater via β-counter with high detector efficiency.

The relative contributions of ^234^Th-^238^U and ^212^Bi-^228^Th should vary with distinct sea areas. The specific character of sediment and intensity of sediment resuspension will determine their relative contributions as well as activity of RA_P234_.

### Seawater turbidity to indicate sediment resuspension

**Figure 2:**
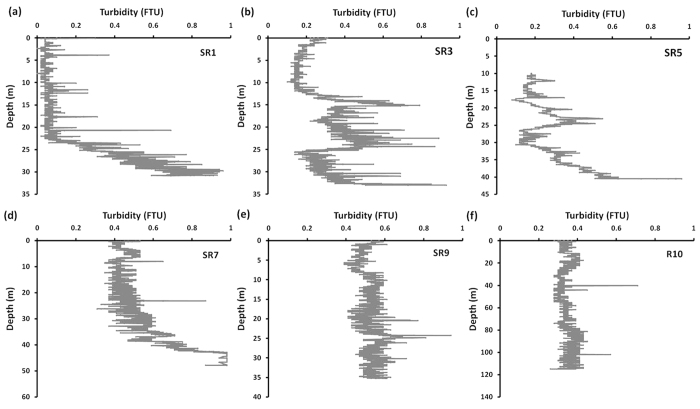
Seawater turbidity at stations SR1 (**a**), SR3 (**b**), SR5 (**c**), SR7 (**d**), SR9 (**e**), R10 (**f**).

Seawater turbidity was measured at six stations ranging in location from the southern Chukchi Sea to the open Arctic Ocean during the CHINARE-6. Extremely high turbidity was observed within the bottom 10 m layer at stations SR1, SR3, SR5, and SR7 (Fig. 2a–d), which were located in the southern and central Chukchi Sea, suggesting that intensive sediment resuspension occurred near the bottom on the shallow continental shelf. This is expected because strong bottom currents in the Chukchi Sea are often observed in summer^15^,^34^. In contrast, seawater turbidity was almost invariable with depth at stations SR9 and R10 (Figs 2e [Fig f3]and 4f), which were located on the northern shelf or in the open ocean. Neither weak currents nor great depth should favor sediment resuspension^15^,^34^.

Long term time series of measurements for ocean currents and other parameters had been collected in the Chukchi Sea^15^,^34^. These observations indicate that there was an active interaction between the sediment and the seawater on the shallow continental shelf, especially during the ice-free summer season. This provides an environmental benefit for generating and sustaining sediment resuspension. An exceptionally strong summer cyclone was reported in early August, 2012 in the Chukchi Sea^35^. The strong mixing and upwelling caused by the cyclone resulted in a relatively well-mixed, vertically homogeneous water column on the continental shelf. Thus, the summer cyclone is another factor that is favourable to the generation of sediment resuspension. Therefore, it is reasonable to infer that sediment resuspension probably took place and redistributed RA_P234_ on the continental shelf.

### Residence time of total ^234^Th to indicate sediment resuspension

The residence time of total ^234^Th was calculated and represented using an irreversible steady-state model (Appendix Table A1)^36^. Our results were consistent with other studies of this region^37^,^38^,^39^. The residence time of total ^234^Th on the continental shelf was significantly shorter than that in the open Arctic Ocean. The high nutrients waters supplied from the North Pacific Ocean can support high photosynthesis and scavenge ^234^Th on the continental shelf relating to nutrient depletion and low photosynthesis in the open ocean^40^,^41^.

The residence time of total ^234^Th for bottom seawater was shorter than that for upper-layer seawater, which had been attributed to sediment resuspension to enhance the scavenging of ^234^Th from seawater^42^. Therefore, the short residence time of total ^234^Th for the bottom layer also provided another clue to sediment resuspension on this shallow, but hydrodynamically active, continental shelf.

### RA_P234_ and POC to indicate sediment resuspension

**Figure 3 f3:**
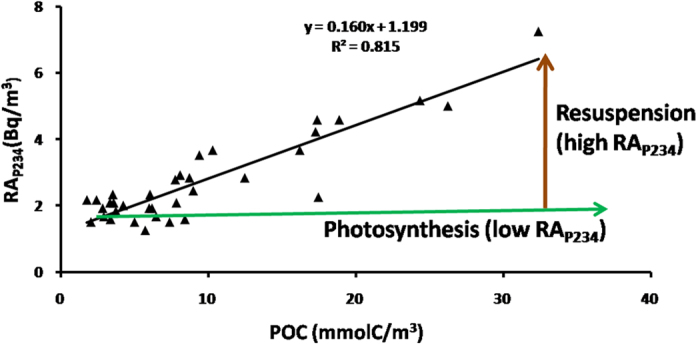
RA_P234_ versus POC in the western Arctic Ocean. The photosynthesis indicated by green arrow can elevate POC but lower RA_P234_. Resuspension is represented by brown arrow and characterized by high RA_P234_.

The relationship between RA_P234_ and POC was investigated in the western Arctic Ocean (Fig. 3). The slope of linear regression line between RA_P234_ and POC was about 0.160 Bq/mmol C for suspended particles. As for the end-member of sediment in the Chukchi Sea, the activity of ^238^U was about 50 Bq/kg^28^, while the activity of ^232^Th was generally comparable with that of ^238^U in the marine sediment^32^. The average concentration of POC was about 1% with a range of 0.5% to 2% in the marine sediment^43^. Thus, the sediment fingerprint is characterized by its ratio of ^238^U-^234^Th and ^232^Th-^228^Th to POC of 0.09 Bq/mmolC with a range of 0.045 to 0.18, which was consistent with the ratio of 0.16 for RA_P234_ to POC (Fig. 3). The linear regression between RA_P234_ and POC also gave a clue to sediment resuspension.

### Conceptual model of RA_P234_

**Figure 4:**
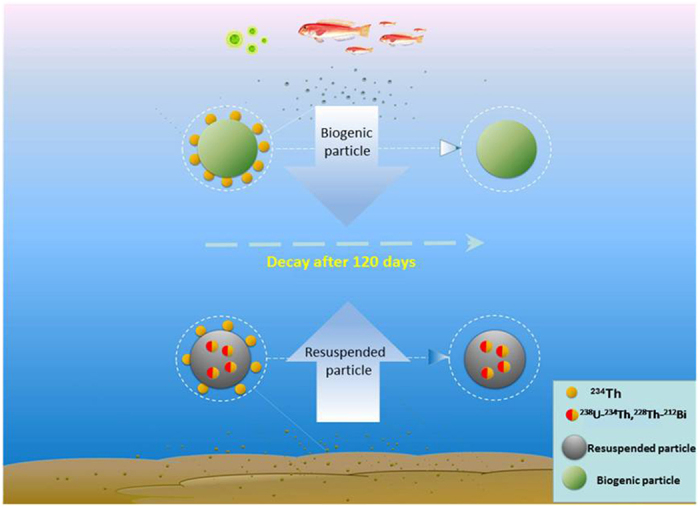
Conceptual model of RA_P234_. The green and grey particles represent biogenic and resuspended particles, respectively. The yellow particles refer to the surface-bound ^234^Th on the particles, which decays away after 120 days. The half-yellow/half-red particles represent internal radionuclides with long half-life parent radionuclides, such as ^234^Th supported by ^238^U and ^212^Bi supported by ^228^Th.

The conceptual model of RA_P234_ is illustrated in Fig. 4. Biogenic and terrigenous particles make the dominant contributions to suspended particles in the upper ocean and bottom nepheloid layer, respectively. Both kinds of particles can adsorb high particle-reactive radionuclides onto particle surfaces. In seawater, the dominant surface-bound radionuclide is ^234^Th. The external and unsupported ^234^Th adsorbed on biogenic and terrigenous particles decays away after 120 days and should not contribute to RA_P234_. Radionuclides with high atomic number are seldom taken up by biotas as essential elements. Thus, biogenic particles play a minor role in RA_P234_. The internal radionuclides of terrigenous particles, dominated by ^234^Th supported by ^238^U and ^212^Bi supported by ^228^Th, still exist and contribute to the second β counting rate of particulate ^234^Th after 120 days due to the long half-lives of ^238^U (4.47 × 10^9^y) and ^228^Th-^232^Th (1.91y and 1.4 × 10^10^y) in the minerals. Both ^228^Th and ^238^U have been categorised as lithophile elements according to Goldschmidt’s classification^44^, which is analogous to aluminium, titanium and other lithophile elements to trace the terrigenous fraction^45^. Although ^228^Th and ^238^U were not measured directly in our study due to the limitation of seawater volume, both ^228^Th and ^238^U in resuspended particles had been directly measured and attributed to sediment resuspension in other studies^29^,^31^.

On the continental shelf, low activity of RA_P234_ in the upper layer and high value in the deep layer at SR3 can be interpreted as dominant photosynthesis and sediment resuspension, respectively. In comparison, low activity of RA_P234_ remained stable considering of its activity uncertainty at SR15 in the open ocean (Fig. 1a), while a peak value of POC was observed in the subsurface layer at a depth of 47 m (Appendix Table A1, 3.57 mmolC/m^3^ at SR15). Subsurface chlorophyll maximum had been widely observed in the Arctic Ocean due to the supplement of nutrients in the subsurface layer^46^. Although POC was variable due to heterogeneous photosynthesis, RA_P234_ was vertically uniform as a result of small contributions to RA_P234_ from biogenic particles.

Consequently, RA_P234_ refers to the terrigenous particles resuspended from marine sediment, which is probable to trace sediment resuspension with sufficient sensitivity via β counter. RA_P234_ could be a nice addition to the ^234^Th/^238^U disequilibrium and seawater turbidity methods to distinguish particle processes related to photosynthesis and sediment resuspension.

### Advantages of RA_P234_

The relationship between RA_P234_ and POC was utilized to distinguish particle processes, including photosynthesis and sediment resuspension, in the western Arctic Ocean (Fig. 3). Biogenic particle were characterized by low RA_P234_ in addition to variable concentrations of POC, which depended on intensity of photosynthesis. In comparison, sediment resuspension can elevate RA_P234_. Therefore, sediment resuspension and photosynthesis could be distinguished with distinct RA_P234_, while seawater turbidity and ^234^Th/^238^U disequilibrium method could not differentiate sediment resuspension from photosynthesis. Additionally, the slope of linear regression between RA_P234_ and POC, ‘slope assumption’, has the potential to indicate the intensity of sediment resuspension (Fig. 3).

**Figure 5:**
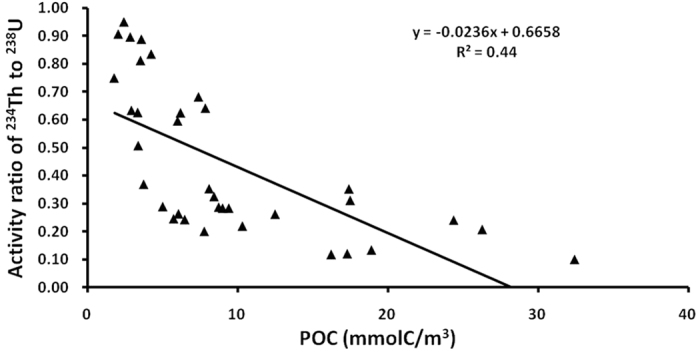
Activity ratio of ^234^Th to ^238^U versus POC in the western Arctic Ocean.

The linear regression between activity ratio of ^234^Th to ^238^U and POC (Fig. 5) was compared with that of RA_P234_ and POC (Fig. 3). The correlation coefficient of RA_P234_ and POC (0.815) is greater than that of ^234^Th/^238^U and POC (0.44). Both sediment resuspension and photosynthesis can enhance the scavenging of ^234^Th. It is difficult to distinguish these two processes via ^234^Th/^238^U method. However, RA_P234_ is directly related to the terrigenous fraction from sediment resuspension based on the conceptual model (Fig. 4). Additionally, the ^234^Th/^238^U disequilibrium method has a memory effect that records the integrated particle dynamics during the past several months^47^. Both RA_P234_ and POC are instantaneous parameters relative to the parameters of ^234^Th/^238^U disequilibrium method with memory effects. Therefore, a better regression result for RA_P234_ and POC was obtained compared with the ^234^Th/^238^U disequilibrium method.

### RA_P234_ and its implications for export flux of ^234^Th

The ^234^Th/^238^U disequilibrium method reflects the integrated particle dynamics, including sediment resuspension and photosynthesis, on the shallow continental shelf. Sediment resuspension can enhance the scavenging of ^234^Th and deficit of ^234^Th to ^238^U, overestimating export flux of ^234^Th. From the conceptual model of RA_P234_, high activity of RA_P234_ was directly related to sediment resuspension. Sediment resuspension can be qualitatively identified on the basis of RA_P234_ to screen out the layer in which sediment resuspension occurred when export flux of ^234^Th was integrated into the shallow water column. However, export flux of ^234^Th may be underestimated following screening when photosynthesis occurs in conjunction with sediment resuspension.

Two endmembers, biogenic particles and resuspended particles, are assumed to exist in bottom seawater. The surface-bound concentrations of ^234^Th on biogenic and resuspended particles were assumed to be f_1_ and f_2_, respectively, in order to estimate the export fluxes of ^234^Th from these two kinds of particles. The exact values of f_1_ and f_2_ were determined by two factors: particle concentration and the adsorbing ability of the particles. Most of the time, the particle concentration could be quantified by chemical proxies with distinct values for biogenic and resuspended particles, such as δ^13^C, Al, Ti and others. Biogenic and resuspended particles have low and high activity of RA_P234_, respectively. Therefore, there is a potential to quantify the concentrations of biogenic and resuspended particles by mean of RA_P234_.

The adsorbing capacity of distinct particle compositions can be quantified by different values of K_d_ for thorium^48^,^49^,^50^. The particle compositions include lithogenic particles, opal, carbonate carbon, organic carbon, etc. If the K_d_ for thorium can be obtained for biogenic and resuspended particles, f_1_ and f_2_ can be calculated (Eqs 1 and 2).


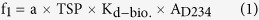



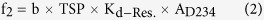


where a and b represent the fraction of biogenic and resuspended particles derived from chemical proxies. TSP is the total suspended particles in the seawater (mg/L). K_d−bio._ and K_d−res._ are the particle-seawater distribution coefficients for biogenic and resuspended particles (L/kg), and A_D234_ is the dissolved activity of ^234^Th in seawater (Bq/m^3^). If f_1_ and f_2_ (Bq/m^3^) can be calculated, the export flux of ^234^Th (F_234−bio._) derived from the biogenic process can be obtained:


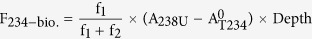


Substituting for f_1_ and f_2_ gives


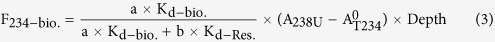


Therefore, the export flux of ^234^Th (F_234−bio._) derived from biogenic process can be determined from the fraction of biogenic particles and K_d_. In natural seawater, the fraction of biogenic particles, along with its uncertainty, can be quantified by chemical proxy. Large uncertainties in the fraction of particles occur, because the chemical proxies for endmember are generally difficult to identify. Although the K_d_ of thorium for distinct particle compositions had been derived under the laboratory conditions^48^,^49^,^50^, K_d−Bio._ and K_d−Res._ are difficult to obtain in natural seawater, especially when complex particle compositions co-occur in biogenic and resuspended particles. The accuracy of the particle fraction and K_d_ will constrain the exact estimation for export fluxes of ^234^Th derived from biogenic particles.

### RA_P234_: a linkage of the atmosphere-ocean-sediment system

**Figure 6:**
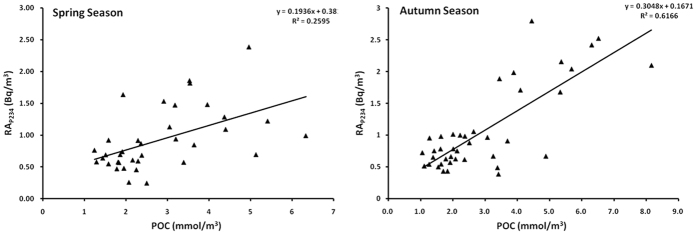
Regression analysis of RA_P234_ and POC in the South China Sea during spring and autumn.

To validate the ‘slope assumption’, the A transect was revisited to investigate RA_P234_ and POC in the South China Sea during spring and autumn. The slope of linear regression between RA_P234_ and POC in Fig. 6 was also greater in autumn (0.30) than in spring (0.19), which may be attributed to sediment resuspension. Sediment resuspension could increase the terrigenous fraction and elevate RA_P234_. The intensity of sediment resuspension had been indicated to be high in the same sea region in autumn relative to spring via the ratio of particulate ^234^Th to dissolved ^234^Th under the influence of the East Asian monsoon system^8^. Therefore, the assumption of the slope of linear regression between RA_P234_ and POC is confirmed in the South China Sea. RA_P234_ will shed new light on ^234^Th-based particle dynamics to investigate the linkage of the atmosphere-ocean-sediment system, such as the typhoons and their impacts on sediment.

A novel approach of RA_P234_ is proposed for the first time to trace sediment resuspension from low- to high-latitude oceans. High activity of RA_P234_ was widely observed on the continental shelf in relation to sediment resuspension (Fig. 1). Sediment resuspension was also corroborated by seawater turbidity, residence time of total ^234^Th, Goldschmidt’s classification, and fingerprint ratio of RA_P234_ to POC from the sediment endmember in the western Arctic Ocean. The mechanism and conceptual model of RA_P234_ was investigated and illustrated (Fig. 4.). RA_P234_ is sufficiently sensitive to identify sediment resuspension via β counter with high detector efficiency. The advantage of RA_P234_ is that it is a supplementary parameter to the ^234^Th/^238^U disequilibrium method and does not require any additional sampling and measurement to distinguish sediment resuspension from photosynthesis, while both the ^234^Th/^238^U disequilibrium and seawater turbidity methods cannot differentiate biogenic particles from terrigenous particles. RA_P234_ is a potential proxy to trace sediment resuspension without a memory effect. RA_P234_ could also be used to screen out the layer to bias integration of ^234^Th and POC fluxes. The slope of the linear regression between RA_P234_ and POC was used to indicate the higher intensity of sediment resuspension in the South China Sea during autumn. Similar to the definition of gross β, RA_P234_ may stimulate some debate but is also meaningful to identify and indicate the intensity of sediment resuspension. From the mechanism proposed, RA_P234_ refers to the terrigenous fraction and has potentially broad implications for investigating the dynamics of suspended particles in the estuary-shelf-slope-ocean continuum and the linkage of the atmosphere-ocean-sediment system.

## Methods

### Sampling

**Figure 7:**
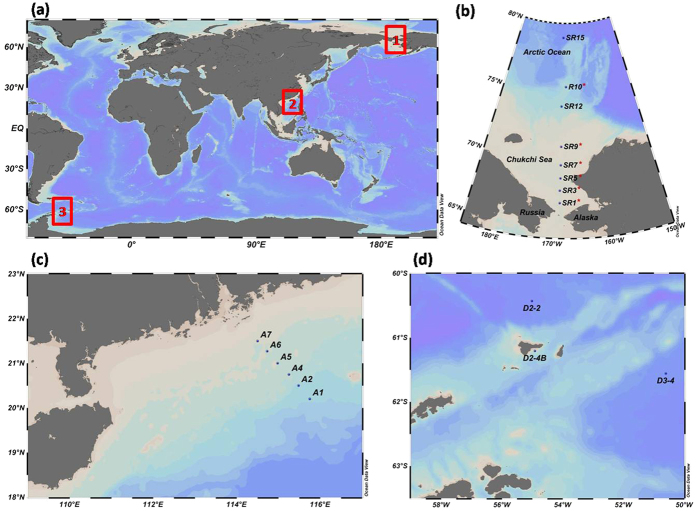
Station map for the western Arctic Ocean (1), the South China Sea (2), and the Southern Ocean (3). These maps were drawn by ODV 4.7.4 (https://odv.awi.de/)^58^.

Seawater samples were collected for ^234^Th analysis from low- to high-latitude ocean in the western Arctic Ocean, the South China Sea, and the Southern Ocean (Fig. 7). Seven stations (SR1, SR3, SR5, SR7, SR9, SR12, SR15) were sampled in the western Arctic Ocean during the 5th Chinese National Arctic Research Expedition (CHINARE-5) in September, 2012 (Fig. 7b.). The sea ice extent during the sampling period was the lowest since the first satellite measurement taken in 1979^51^. Seawater turbidity was measured and was indicated by red stars on the continental shelf (SR1, SR3, SR5, SR7, SR9) and the open ocean (R10) (Fig. 7b).

A transect (six stations) was taken from the continental shelf to the open ocean outside the mouth of the Pearl River in the northern South China Sea during 2–8 November, 2010 (autumn) and 16–18 May, 2011 (spring)^8^ (Fig. 7c). Stations A7, A6, and A5 were on the continental shelf (depth < 100 m). Three stations were analysed around Elephant Island, off the north-eastern Antarctic Peninsula on 22–25 January, 2012 during the 28th CHINARE-Antarctic (Fig. 7d). Only station D2-4B was near coast of Elephant Island with the depth of 53 m. Two stations (D2-2 and D3-4) were in the open ocean with the depth over 3000 m.

### Analysis of ^234^Th

The ^234^Th/^238^U disequilibrium method has been widely applied in the global ocean with a huge database to quantify the marine biological carbon pump^52^, which modulates glacial/interglacial atmospheric carbon dioxide and climate change^53^. The international calibration of ^234^Th was conducted under the framework of GEOTRACES^54^. The small-volume technique via β counting of ^234^Th has been extensively studied due to its high sampling resolution^13^,^23^. The radiochemical analysis of ^234^Th had been described^8^,^37^.

Following filtration of seawater with 25-mm diameter Quartz Microfiber (QMA, nominal pore size 1.0 μm), the direct measurement of particulate ^234^Th without radiochemical separation was obtained from the difference in values between the first β counting after sampling and the second β counting after 120 days as a result of high activity of ^234^Th in seawater^19^. After treating with MnO_2_ co-precipitation, the activity of total ^234^Th was also calculated from the difference between the first and second β counting rates of total ^234^Th. The activity of ^234^Th and its associated uncertainty were calculated according to Eqs 4, 5, 6, 7, 8, 9.


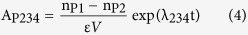



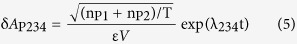



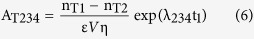



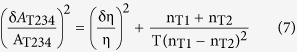



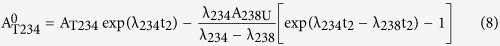



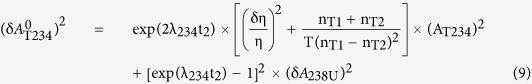


The dimensions and definitions of the parameters are given in Table 4. Equations 4, 5, 6, 7, 8, 9 had been deduced in detail with the similar principle^55^.

### Definition and calculation of RA_P234_

The second counting rate of particulate ^234^Th (n_P2_) was usually overlooked, because only the difference between the first and second counting rates (n_P1_-n_P2_) was used to calculate particulate ^234^Th using Eq. 4. In the open ocean, the second counting rate of particulate ^234^Th (n_P2_) was relatively stable with a value of 0.3~0.4 cpm, which also depends on the instrumental background with a normal value of 0.15~0.2 cpm via gas-flow proportional low-level RISØ β-counter (Model GM-25-5, RISØ National Laboratory, Denmark)^12^,^13^,^14^. In this study, the abnormally high second counting rate of particulate ^234^Th was observed for bottom seawater on the continental shelf in the western Arctic Ocean. This phenomenon was further confirmed in the South China Sea and the Southern Ocean. RA_P234_ derived from the second counting rate of particulate ^234^Th and instrumental background was proposed for the first time to investigate this abnormal value of particulate ^234^Th. Equations 10 and 11 were used to calculate RA_P234_ and its uncertainty:


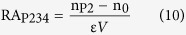



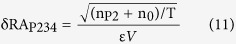


All the parameters in Eqs 10 and 11 are defined in Table 4. The detector efficiency of RA_P234_ is equal to that of particulate ^234^Th because of the similar energy of β particles being emitting by radionuclide candidates. The second counting rate of particulate ^234^Th (0.54 ± 0.02 cpm) was very constant after 136 days, 304 days, and 495 days from the sampling date, which indicates that it was mainly radionuclides with long half-lives that contributed to RA_P234_. The stability of the second counting rate of particulate ^234^Th has been demonstrated^24^.

Notice that RA_P234_ was not a signal from a certain radionuclide. In fact, RA_P234_ was the residual β activity for particulate ^234^Th after more than 120 days, which was usually recognized to be the stable methodological background for particulate ^234^Th and was therefore neglected. This residual β activity may include several radionuclides with long half-lives. It should be treated as a supplementary parameter for total ^234^Th and particulate ^234^Th and has advantage of being able to trace sediment resuspension without any additional sampling and analysis based on the small-volume technique for ^234^Th.

The definition of RA_P234_ is similar to that of gross β in drinking water. Most of the time, the exact radionuclides and their contributions to gross β cannot be identified^11^. However, gross β is an essential parameter for screening the level of radiological pollution, especially during nuclear emergency. The detector efficiencies of ^90^Sr and ^137^Cs are artificially chosen to calculate that of gross β for drinking water, although a spread of energies of β particles from distinct radionuclides (^40^K) with different detector efficiencies is very common^11^. Analogously, the definition of RA_P234_ is proposed and is convenient for tracing sediment resuspension without any additional sampling and measurement.

Although the abnormally high second counting rate of total ^234^Th was also observed for the bottom seawater on the continental shelf as that of particulate ^234^Th, the second counting rate of total ^234^Th was not discussed in this study. The radionuclides contributing to the second counting rate of total ^234^Th are more complex than that of particulate ^234^Th due to the additional MnO_2_ co-precipitation. The radiochemical treatment of MnO_2_ co-precipitation for total ^234^Th can scavenge other radionuclides of low K_d_ with variable chemical recovery, such as radium and its progenies, onto the MnO_2_-particle surface^13^,^56^, especially for the tectonically active sea region with ^224^Ra diffusion into the overlying seawater^25^.

### Particulate organic carbon

Following the second counting of particulate ^234^Th, the POC was measured with an Elemental Analyzer (Elementar vario EL III) after removing the carbonate fraction by fuming with concentrated hydrochloric acid^57^. The blank of the method was subtracted. The analytical precision was always better than 10%.

### Seawater turbidity

The seawater turbidity was measured using a turbidity sensor (Rinko-profiler) during the 6th CHINARE from 27 July to 7 August 2014. The turbidity sensor works on the basis of backscattering principle and has a range of 0~1 FTU. The reference material was Formazin. A few abnormal values over 1 FTU arising from the present of bubbles were discarded. The precision of the turbidity sensor was 0.03 FTU.

## Additional Information

**How to cite this article**: Lin, W. *et al*. Residual β activity of particulate ^234^Th as a novel proxy for tracking sediment resuspension in the ocean. *Sci. Rep.* **6**, 27069; doi: 10.1038/srep27069 (2016).

## Supplementary Material

Supplementary Information

## Figures and Tables

**Table 1:** Typical activities of radionuclides in natural seawater.

**Radionuclide (Low K**_**d**_)	**Activity (Bq/m**^**3**^)	**Radionuclide (High K**_**d**_)	**Activity (Bq/m**^**3**^)
^40^K	11,000~12,000	^234^Th	44
^87^Rb	110	^210^Pb, ^210^Bi, ^210^Po	1~10
^3^H	70	^227^Ac, ^228^Th, ^230^Th, ^231^Pa^239+240^Pu, ^241^Am	10^−3^~10^−2^
^234^U	48		
^238^U	44	^232^Th	10^−4^
^14^C	6		
^235^U	2.2		
^7^Be, Ra, Rn, ^90^Sr, ^37^Cs	1~10		
^32^P, ^33^P	10^−1^		
^32^Si, ^99^Tc	10^−3^~10^−2^		
^10^Be, ^35^S, ^37^Ar, ^39^Ar	10^−4^		
^129^I	10^−5^		

**Table 2:** Activity, emitting particle (with energy), and radionuclide yield in marine sediments^59^.

**Radionuclide**	**Typical activity (Bq/kg-d.w.)**	**Emitting particle**	**Energy (MeV)**	**Yield (%)**
^40^K	500	β	1.31	89.3
^210^Pb	150	β	0.063	19.8
^210^Bi	150	β	1.16	100
^210^Po	150	α	5.30	100
^230^Th	150	α	4.69	76.3
^87^Rb	120	β	0.273	100
^7^Be	100	γ	0.48	10.4
^226^Ra	10–100	α	4.78	94.5
^228^Ra	10–100	β	0.039	100
^228^Ac	10–100	β	1.17	32
			1.74	12
^228^Th	10–100	α	5.42	72.7
^212^Bi	10–100	β	2.25	48.4
^232^Th	10–100	α	4.01	77
^238^U	10–100	α	4.20	77
^234^U	10–100	α	4.77	72.4
^234^Th	10–100	β	0.19	72.5
^234m^Pa	10–100	β	2.28	99
^14^C	1–10	β	0.15	100
^90^Sr	1–10	β	0.55	100
^90^Y	1–10	β	2.28	100
^137^Cs	1–10	β	0.51	94.6
^235^U	1–10	α	4.40	55
^239+240^Pu	0.1–1	α	5.1	100
^241^Am	0.1–1	α	5.4	100
^3^H	10^−2^	β	0.018	100

**Table 3:** Typical activities of radionuclides in marine biotas (in Bq/kg-f.w.)^59^,^60^.

**Radionuclide**	**Phytoplankton**	**Zooplankton**	**Flatfish**	**Crab**	**Seaweed**	**Fish**
^40^K	100	100	82.9	71.9	370	83
^14^C	ND^a^	ND	19	16	14	19
^210^Po	2.3	25	15	15	2	30
^228^Ra	ND	ND	1.8	1.8	2.2	1.8
^210^Pb	ND	ND	0.28	0.49	2.2	0.39
^226^Ra	2.7	0.2	0.11	5.9 × 10^−2^	2	0.4
^87^Rb	ND	ND	0.74	0.94	4.6	0.72
^3^H	ND	ND	5 × 10^−2^	5 × 10^−2^	5 × 10^−2^	5 × 10^−2^
^228^Th	1	0.3	2.5 × 10^−2^	5.5 × 10^−2^	1.2	3 × 10^−2^
^232^Th	0.1	0.06	1.1 × 10^−3^	1.1 × 10^−2^	0.31	1.3 × 10^−3^
^234^Th, ^234^U, ^238^U	0.4	0.2	2.5 × 10^−3^	1.3 × 10^−1^	1.7	5.6 × 10^−3^
^230^Th	0.1	0.06	9.5 × 10^−4^	6.2 × 10^−2^	0.11	3.1 × 10^−3^
^235^U	ND	ND	1.1 × 10^−4^	5 × 10^−3^	7.3 × 10^−2^	3.6 × 10^−4^

^a^“ND” denotes no data.

**Table 4:** List of parameters with dimensions and definitions.

**Parameter**	**Dimension**	**Definition**
Constants		
λ_234_	day^−1^	Decay constant of ^234^Th
λ_238_	day^−1^	Decay constant of ^238^U
Sample information		
t	day	Elapsed time between sampling date and detecting date
t_1_	day	Elapsed time between MnO_2_ formation date and detecting date
t_2_	day	Elapsed time between sampling date and MnO_2_ formation date
T	min	β-counting time for particulate and total ^234^Th
V	m^3^	Volume of seawater
Detector information		
ε		Detector efficiency
Processing and analysis information		
n_P1_	Bq	First β counting rate of particulate ^234^Th
n_P2_	Bq	Second β counting rate of particulate ^234^Th
n_T1_	Bq	First β counting rate of total ^234^Th
n_T2_	Bq	Second β counting rate of total ^234^Th
n_0_	Bq	Instrumental background with a normal value of 0.2 cpm
η		Chemical recovery of ^230^Th
δη		Uncertainty of η
Calculated parameter		
A_P234_	Bq/m^3^	Activity of particulate ^234^Th at sampling time
A_T234_	Bq/m^3^	Activity of total ^234^Th at MnO_2_ formation time
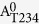	Bq/m^3^	Activity of total ^234^Th at sampling time
A_238U_	Bq/m^3^	Activity of ^238^U at sampling time
RA_P234_	Bq/m^3^	Activity of Residual β activity of particulate ^234^Th at sampling time
δA_P234_		Uncertainty of particulate ^234^Th
δA_T234_		Uncertainty of A_T234_
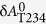		Uncertainty of 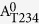
δA_238U_		Uncertainty of A_238U_
δRA_P234_		Uncertainty of RA_P234_
