# Supplementary material for: Residual β activity of particulate 234Th as a novel proxy for tracking sediment resuspension in the ocean
Source: Sci Rep. 2016 Jun 2;6:27069. doi: 10.1038/srep27069 (PMC4890035; doi:10.1038/srep27069)
Supplement: Supplementary Information [file srep27069-s1.doc]

**Residual β activity of particulate ^234^Th as a novel proxy for tracking sediment resuspension in the ocean**

Wuhui Lin^1^, Liqi Chen^2*^, Shi Zeng^3^, Tao Li^4^, Yinghui Wang^1^, Kefu Yu^1^

^1^ School of Marine Science, Guangxi University, Nanning, China

^2^ Key Laboratory of Global Change and Marine-Atmospheric Chemistry, Third Institute of Oceanography, State Oceanic Administration, Xiamen, China

^3^ Department of Engineering Physics, Tsinghua University, Beijing, China

^4^ Ocean University of China, Qingdao, China

**Correspondence to:**

Liqi Chen,E-mail: [chenliqi@tio.org.cn](mailto:chenliqi@tio.org.cn); Wuhui Lin, E-mail: [linwuhui8@163.com](mailto:linwuhui8@163.com)

Add. Key Laboratory of Global Change and Marine-Atmospheric Chemistry, State Oceanic Administration, 361005, China. Tel./fax: +86 592 2195353.

**Appendix**

Table A.1 The activity of RA_P234_, ^234^Th, ^238^U, POC concentration, and residence time of total ^234^Th in the western Arctic Ocean. The activity uncertainty of RA_P234_ was represented with the confidence level of 63.7% (k=1).

| **Station** | **Depth (m)** | **RA_P234_**  **(Bq/m^3^)** | **POC**  **(mmol C/m^3^)** | **A_T234_^0^**  **(Bq/m^3^)** | **A_238U_**  **(Bq/m^3^)** | **τ_T234_**  **(day)** |
| --- | --- | --- | --- | --- | --- | --- |
| SR1  (Bottom depth,42m) | 8 | 2.78±0.34 | 7.75 | 6.92±0.94 | 34.53±1.03 | 8.72 |
|  | 10 | 1.25±0.23 | 5.71 | 8.48±0.92 | 34.53±1.03 | 11.33 |
|  | 20 | 2.44±0.33 | 8.97 | 10.79±0.90 | 38.07±1.14 | 13.76 |
|  | 31 | 2.83±0.27 | 8.71 | 10.96±0.92 | 38.20±1.15 | 13.99 |
|  | 38 | 3.67±0.36 | 10.29 | 8.37±0.89 | 38.20±1.15 | 9.75 |
| SR3  (Bottom depth,51m) | 4 | 2.25±0.26 | 17.45 | 10.26±0.80 | 32.97±0.99 | 15.72 |
|  | 10 | 1.92±0.25 | 6.17 | 24.24±0.95 | 38.80±1.16 | 57.89 |
|  | 21 | 4.58±0.31 | 17.37 | 13.76±0.92 | 39.10±1.17 | 18.89 |
|  | 31 | 5.17±0.32 | 24.34 | 9.41±0.87 | 39.13±1.17 | 11.02 |
|  | 46 | 5.00±0.31 | 26.23 | 8.11±0.89 | 39.14±1.17 | 9.09 |
| SR5  (Bottom depth,52m) | 6 | 1.58±0.24 | 8.40 | 10.53±0.77 | 32.40±0.97 | 16.73 |
|  | 10 | 2.92±0.27 | 8.08 | 13.30±0.81 | 37.68±1.13 | 18.98 |
|  | 20 | 4.22±0.37 | 17.26 | 4.61±0.80 | 38.30±1.15 | 4.76 |
|  | 31 | 4.58±0.31 | 18.87 | 5.12±0.76 | 38.33±1.15 | 5.37 |
|  | 50 | 7.25±0.35 | 32.37 | 3.82±0.79 | 38.35±1.15 | 3.85 |
| SR7  (Bottom depth,52m) | 5 | 3.52±0.54 | 9.38 | 9.55±1.96 | 33.69±1.01 | 13.76 |
|  | 10 | 1.67±0.24 | 6.45 | 8.17±0.63 | 33.70±1.01 | 11.13 |
|  | 20 | 2.33±0.26 | 6.04 | 8.92±0.75 | 33.90±1.02 | 12.41 |
|  | 32 | 1.50±0.24 | 4.99 | 10.67±0.81 | 36.93±1.11 | 14.13 |
|  | 51 | 3.67±0.29 | 16.19 | 4.47±0.82 | 37.99±1.14 | 4.63 |
| SR9  (Bottom depth,44m) | 5 | 2.08±0.25 | 7.82 | 24.12±0.83 | 37.61±1.13 | 62.23 |
|  | 11 | 1.50±0.24 | 7.37 | 25.64±0.86 | 37.61±1.13 | 74.46 |
|  | 21 | 1.92±0.25 | 5.98 | 22.47±0.84 | 37.72±1.13 | 51.26 |
|  | 31 | 1.83±0.25 | 3.72 | 14.34±0.74 | 38.87±1.17 | 20.33 |
|  | 42 | 2.83±0.27 | 12.46 | 10.18±0.73 | 38.89±1.17 | 12.33 |
| SR12  (Bottom depth,175m) | 7 | 2.08±0.25 | 3.33 | 19.48±0.80 | 31.11±0.93 | 58.22 |
|  | 22 | 2.33±0.26 | 3.51 | 28.23±0.87 | 34.76±1.04 | 150.36 |
|  | 30 | 2.00±0.25 | 4.22 | 30.07±0.90 | 35.99±1.08 | 176.33 |
|  | 50 | 1.67±0.24 | 2.91 | 23.83±0.82 | 37.62±1.13 | 60.12 |
|  | 102 | 1.58±0.24 | 3.36 | 19.70±0.79 | 38.81±1.16 | 35.83 |
| SR15  (Bottom depth,3068m) | 7 | 1.50±0.24 | 2.03 | 28.93±0.92 | 31.89±0.96 | 339.44 |
|  | 22 | 2.17±0.26 | 2.41 | 30.34±0.92 | 31.92±0.96 | 668.19 |
|  | 31 | 1.92±0.25 | 2.83 | 31.98±0.95 | 35.68±1.07 | 300.47 |
|  | 47 | 2.08±0.25 | 3.57 | 32.71±0.94 | 36.83±1.10 | 276.53 |
|  | 104 | 2.17±0.26 | 1.76 | 28.64±0.89 | 38.21±1.15 | 104.07 |

Table A.2 The activity of RA_P234_ and concentration of POC for the suspended particle in the South China Sea during the spring and autumn season. The activity uncertainty of RA_P234_ was represented with the confidence level of 63.7% (k=1).

| **Station** | **Depth (m)** | **Autumn Season** | | **Spring Season** | |
| --- | --- | --- | --- | --- | --- |
|  |  | **RA_P234_**  **(Bq/m^3^)** | **POC**  **(mmol C/m^3^)** | **RA_P234_**  **(Bq/m^3^)** | **POC**  **(mmol C/m^3^)** |
| A7  (Bottom depth,73m) | 5 | 2.10±0.26 | 8.16 | 0.94±0.21 | 3.2 |
|  | 25 | 2.52±0.26 | 6.52 | 1.86±0.35 | 3.53 |
|  | 50 | 2.04±0.24 | 5.68 | 1.22±0.25 | 5.41 |
|  | 68 | 2.42±0.25 | 6.30 | 1.29±0.23 | 4.37 |
| A6  (Bottom depth,90m) | 5 | 2.16±0.36 | 5.36 | 1.64±0.23 | 1.93 |
|  | 25 | 1.68±0.26 | 5.33 | 0.57±0.20 | 3.39 |
|  | 50 | 1.71±0.24 | 4.09 | 1.53±0.35 | 2.91 |
|  | 75 | 1.99±0.24 | 3.88 | 1.82±0.26 | 3.54 |
|  | 85 | 2.80±0.26 | 4.44 | 2.39±0.25 | 4.96 |
| A5  (Bottom depth,105m) | 5 | 1.89±0.35 | 3.44 | 1.47±0.22 | 3.18 |
|  | 25 | 0.88±0.23 | 2.51 | 1.13±0.22 | 3.05 |
|  | 50 | 1.05±0.22 | 2.64 | 1.48±0.35 | 3.96 |
|  | 75 | 0.96±0.21 | 3.07 | 1.09±0.24 | 4.4 |
|  | 95 | 0.91±0.21 | 3.69 | NS^1^ | NS^1^ |
| A4  (Bottom depth,188m) | 5 | 0.75±0.17 | 2.13 | 0.25±0.10 | 2.5 |
|  | 25 | 0.43±0.12 | 1.83 | 0.99±0.12 | 6.33 |
|  | 50 | 0.50±0.11 | 1.55 | 0.46±0.10 | 2.25 |
|  | 75 | 0.62±0.11 | 2.08 | 0.92±0.18 | 2.29 |
|  | 100 | 0.57±0.11 | 1.91 | 0.74±0.12 | 1.91 |
|  | 125 | 1.01±0.18 | 2.00 | 0.61±0.11 | 2.16 |
|  | 150 | 0.78±0.13 | 1.61 | NS^1^ | NS^1^ |
| A2  (Bottom depth,395m) | 5 | 0.38±0.11 | 3.41 | 0.69±0.11 | 5.13 |
|  | 25 | 0.67±0.11 | 3.25 | 0.47±0.10 | 1.78 |
|  | 50 | 0.49±0.11 | 3.38 | 0.57±0.17 | 1.83 |
|  | 75 | 0.75±0.17 | 1.42 | 0.59±0.12 | 2.29 |
|  | 100 | 0.54±0.12 | 1.26 | 0.58±0.11 | 1.29 |
|  | 125 | 0.72±0.12 | 1.05 | 0.87±0.11 | 2.36 |
|  | 150 | 1.00±0.12 | 2.22 | 0.55±0.11 | 1.58 |
|  | 200 | 0.78±0.11 | 2.01 | NS^1^ | NS^1^ |
|  | 300 | 0.95±0.18 | 1.27 | NS^1^ | NS^1^ |
| A1  (Bottom depth,743m) | 5 | 0.67±0.11 | 4.88 | 0.69±0.17 | 1.86 |
|  | 25 | 0.62±0.11 | 2.36 | 0.68±0.12 | 2.38 |
|  | 50 | 0.98±0.18 | 2.37 | 0.26±0.10 | 2.07 |
|  | 75 | 0.66±0.12 | 1.94 | 0.85±0.11 | 3.64 |
|  | 100 | 0.43±0.11 | 1.70 | 0.48±0.10 | 1.95 |
|  | 125 | 0.54±0.11 | 1.63 | 0.76±0.17 | 1.24 |
|  | 150 | 0.51±0.11 | 1.11 | 0.69±0.12 | 1.51 |
|  | 200 | 0.98±0.22 | 1.63 | 0.64±0.11 | 1.44 |
|  | 300 | 0.63±0.13 | 1.78 | 0.92±0.12 | 1.58 |
|  | 500 | 0.65±0.11 | 1.38 | 0.58±0.11 | 1.81 |

1. The “NS” denotes the depth was not sampled.

Table A.3 The activity of RA_P234_ and concentration of POC for the suspended particle in the Southern Ocean. The activity uncertainty of RA_P234_ was represented with the confidence level of 63.7% (k=1).

| **Station** | **Depth (m)** | **RA_P234_ (Bq/m^3^)** | **POC (mmol C/m^3^)** |
| --- | --- | --- | --- |
| D2-4B  (Bottom depth, 53m) | 5 | 1.33±0.26 | 10.11 |
|  | 10 | 2.75±0.29 | 10.13 |
|  | 20 | 2.42±0.28 | 9.82 |
|  | 30 | 2.17±0.27 | 9.23 |
|  | 45 | 1.75±0.27 | 8.89 |
| D2-2  (Bottom depth,3535m) | 5 | 1.25±0.25 | 8.07 |
|  | 25 | 1.67±0.26 | 7.11 |
|  | 50 | 1.42±0.26 | 3.23 |
|  | 75 | 1.58±0.26 | 2.38 |
|  | 100 | 1.50±0.26 | 2.13 |
|  | 150 | 1.25±0.25 | 1.68 |
| D3-4  (Bottom depth,3187m) | 5 | 1.25±0.25 | 5.97 |
|  | 25 | 1.33±0.26 | 5.35 |
|  | 50 | 1.83±0.27 | 3.79 |
|  | 75 | 1.67±0.26 | 3.86 |
|  | 100 | 1.33±0.26 | 2.66 |
